# Prehospital hypothermia

**DOI:** 10.1186/cc11261

**Published:** 2012-06-07

**Authors:** Hans-Jörg Busch, Katrin Fink

**Affiliations:** 1 Emergency Department, University Hospital Freiburg, Germany; 2 Department of Cardiology and Angiology, University Hospital Freiburg, Germany

## 

Mild hypothermia is widely used in the treatment of successfully resuscitated patients after cardiac arrest [1]. Previous experimental and clinical studies have demonstrated beneficial effects of cooling after cardiac arrest. Two clinical landmark studies in 2002 demonstrated the use of therapeutic hypothermia after cardiac arrest due to ventricular fibrillation decreases mortality and improves neurological outcome [2,3]. This led the International Liaison Committee on Resuscitation and the American Heart Association to recommend the use of therapeutic hypothermia after cardiac arrest as soon as possible after the return of spontaneous circulation (ROSC) [4].

Despite major progress in intensive care medicine in the last decades, mortality rates after cardiac arrest remain unacceptably high [2,3]. The high mortality rates after cardiac arrest can be attributed to a unique pathophysiological process [1,5,6]. The entity of the pathophysiological changes after ROSC - for example, activation of the inflammatory system - can be summarized as the post-cardiac arrest syndrome [1,5-7].

Hypoxic encephalopathy, which is often a result of the initial hypoxic phase and/or the post-cardiac arrest syndrome, is one of the main causes for mortality, disability and a need for permanent care in patients after cardiac arrest [1].

Pathophysiologically, the resuscitation period could be divided into different time periods. After cessation of circulation, ischemia of different tissues leads to necrotic cell death (hypoxia-induced cellular dysfunction) [7,8]. Reperfusion injury then follows after an imprecise period of time once oxygenated blood is returned to the ischemic tissues with the beginning of mechanical resuscitation (reperfusion-induced cell death) [7,8]. From experimental and clinical studies, it is clear that the tissue damage due to reperfusion occurs over several hours to days in the post-resuscitation phase [1,7,8].

Several experimental studies have emphasized induction of therapeutic hypothermia as soon as possible after ROSC or during cardiopulmonary resuscitation [7-10]. These studies in the different animal models demonstrate a beneficial effect, including attenuation of the cerebral injury after prolonged ischemia due to earlier cooling [7-10]. Recent experimental data in different animal models of cardiac arrest, stroke and myocardial infarction suggest that warm reperfusion under normal or hyperthermic conditions could increase the deleterious effects of the reperfusion. For the effective prevention and treatment of the reperfusion injury, reperfusion should occur in temperature-controlled or cooled tissues.

Nevertheless, prehospital induction of therapeutic hypothermia is still under discussion; consistent protocols are not present and human data are rare. In a retrospective clinical study, early achievement of the target temperature appeared to reduce hypoxic brain injury and favor a good neurologic outcome after successful resuscitation [11].

On the other hand, a small retrospective, observational investigation found a faster decline in body temperature to the target temperature is linked to a less favorable neurologic outcome in comatose patients after cardiac arrest treated with therapeutic hypothermia [12]. However, this may simply indicate a severe ischemic damage with consecutive impaired thermoregulation [12].

In the PRINCE study, feasibility of preclinical transnasal cooling with evaporated perfluorcarbon that primarily leads to a prior selective cooling of the cerebrum was analyzed. In a subgroup of patients, intra-arrest hypothermia via evaporated perfluorcarbon was beneficial [13,14]. Several other studies show also safety and feasibility of prehospital hypothermia [15,16]. In summary, prehospital treatment of patients with a cardiac cause of the arrest may increase the rate of favorable outcome at hospital discharge. Further larger clinical investigations are needed to evaluate the effects of prehospital cooling in cardiac arrest patients [7,8]. In a small survey of emergency physicians in Germany, only a minority of patients is frequently treated with hypothermia before hospital admission after successful resuscitation [7,8].

However, taking the pathophysiological processes into consideration, induction of therapeutic hypothermia should not be limited to the ICUs but should also be able in the field or in the emergency department. Different methods are available to achieve and maintain the target temperature in the prehospital setting [7,8].
